# Leaflet: Operative Steps for Interventional Studies in Neuroscience

**DOI:** 10.3390/neurolint17010001

**Published:** 2024-12-24

**Authors:** Maria Meringolo, Sergio Delle Monache, Giuseppina Martella, Antonella Peppe

**Affiliations:** 1 Santa Lucia Foundation, IRCCS Fondazione Santa Lucia, 00179 Rome, Italy; m.merigolo@hsantalucia.it (M.M.); a.peppe@hsantalucia.it (A.P.); 2 Faculty of Medicine and Surgery, Saint Camillus International University of Health and Medical Sciences, 00131 Rome, Italy; 3 Department of Systems Medicine and Centre of Space Bio-Medicine, University of Rome Tor Vergata, 00133 Rome, Italy; 4 Department of Psychology and Health Sciences, Faculty of Humanities Educations and Sports, Pegaso Telematics University, 80143 Naples, Italy

**Keywords:** clinical trials, study design, law procedures, statistical approach, health, patients, non-commercial clinical trials, enrollment, regulatory agencies, packaging

## Abstract

Background/Objectives: Drug development involves multiple stages, spanning from initial discovery to clinical trials. This intricate process entails understanding disease mechanisms, identifying potential drug targets, and evaluating the efficacy and safety of candidate drugs. Clinical trials are designed to assess the effects of drugs on humans, focusing on determining safety profiles, appropriate modes of administration, and comparative efficacy against placebos. Notably, neuroscience drug development encounters distinct challenges, including the complex nature of diseases, limitations imposed by the blood–brain barrier, the absence of reliable predictive preclinical models, and regulatory hurdles. Ethical and safety considerations are pivotal due to the potential cognitive and motor effects of CNS-active drugs. Methods: Our manuscript outlines the procedures for CNS clinical trials and highlights the key elements of study design, methodological considerations, and ethical frameworks. To achieve our objectives, we considered the official websites of regulatory authorities, the EQUATOR network, and recent publications in the field. The paper includes key elements such as criteria for subject selection, methods of evaluation, variable analysis, and statistical methodology approaches. Results: We want to furnish a concise and comprehensive guide tailored to individuals new to CNS clinical trials, providing foundational elements necessary for the design and execution of such trials. The manuscript seeks to outline sources of relevant materials and elucidate adaptability, particularly in instances where sponsors may be absent. Conclusions: By meeting the needs of less-experienced researchers or those with limited resources, the intention is to facilitate an understanding of the intricate nature of the process and offer guidance on appropriately navigating its complexities. It is essential to note that this manuscript does not aim to be exhaustive but endeavors to serve as a structured checklist. Through its approach, the manuscript aspires to offer guidance and support to individuals navigating the challenges inherent in this intricate domain.

## 1. Introduction

**Figure 1:**
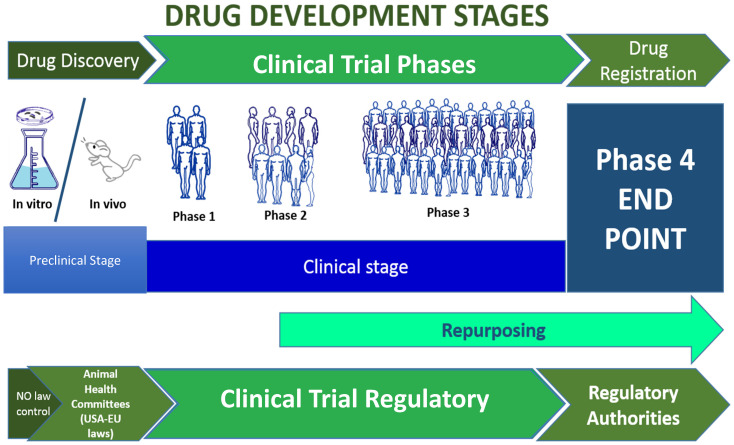
Drug development and clinical trial stages.

Developing a new drug involves several key stages: discovery, preclinical development, and clinical trials (Figure 1 and Supplemental Materials). Initially, research focuses on understanding fundamental molecular mechanisms underlying diseases and identifying potential molecular targets for developing new drug candidates. During the preclinical development phase, the mechanism of action of these candidate drugs is elucidated, and potential toxicity and efficacy are evaluated using in vitro and in vivo models. The transition from the discovery phase to preclinical development is a continuous process, where initial pharmacological and toxicological testing results often guide the selection of the primary drug candidate (see Supplemental Materials).

Clinical trials involving human subjects aim to investigate various aspects of the drug candidate. These trials assess the drug’s side effects, propose suitable modes of administration, evaluate safety and efficacy under specific health conditions, and compare its effects to those of a placebo [1,2,3,4]. The demarcation between preclinical development and clinical trials is established by submitting a new drug application, which is a requisite step to initiate clinical trials. Finally, subsequent stages encompass review, approval, and post-marketing monitoring. These phases determine whether the drug is approved for general use and oversee its performance post-launch, assessing long-term risks and benefits in real-world scenarios [1,5].

The drug development process is an extensive and complex journey that typically spans 15–20 years from discovery to market approval. This process is divided into several key stages, each serving a distinct purpose in ensuring a potential therapeutic’s safety, efficacy, and overall success. Between 1–2 and 6 years are required in the preclinical stage. In this stage, drug candidates undergo extensive in vivo testing in animal models to assess safety, toxicity, pharmacokinetics (how the drug is absorbed, distributed, metabolized, and excreted), and preliminary efficacy. Then, taking from 3 to 10 years, it is necessary to develop the central clinical stages (Phases 1–3). These phases focus on the safety, efficacy, dosage evaluations, and the required extensive data collection. Phase 4 becomes active after the drug has been approved for 10–20 years [6].

Developing new drugs for central nervous system (CNS) disorders in neuroscience faces unique challenges. These include the increased complexity and heterogeneity of diseases, the presence of a blood–brain barrier that limits the flow of molecules, a lack of relevant preclinical models with predictive validity, patient/phenotype heterogeneity, efficacy and safety issues, lack of biomarkers, high placebo effects, insufficient CNS protocols, ethical considerations, and regulatory challenges [7].

Preclinical and clinical studies conducted on CNS-active molecules are generally more detailed and rigorous because of their potential effect on cognitive and motor abilities and the potential for abuse, physical dependence, withdrawal, rebound, and additive effects [8]. Furthermore, in addition to meeting general regulatory guidelines for clinical trials involving investigational new drugs, submission of CNS-active drugs may require additional considerations and evaluations.

In 2016, an examination conducted by the European Medicines Agency (EMA) of 103 submissions in the fields of neurology and psychiatry, spanning from 1995 to 2014, brought attention to significant concerns regarding the effectiveness and safety of treatments [8]. The analysis revealed that approximately one-third of these submissions exhibited efficacy issues, while more than half raised safety concerns. Notably, over 50% of the applications experiencing major problems with their intended outcomes had faced difficulties in their initial clinical development stages. In contrast, only 13% of the submissions without such concerns encountered similar challenges. Moreover, a staggering 91% of the submissions encountered issues during the early phases of development, and they experienced hurdles in conducting subsequent confirmatory studies. Additionally, the examination pointed out distinct challenges between neurology and psychiatry medicines. Specifically, psychiatry-related submissions displayed more pronounced concerns regarding planning and clinical relevance during confirmatory studies, significantly impacting the overall outcomes. Consequently, in response to these findings, the EMA made the decision to develop and publish scientific guidelines specifically aimed at evaluating drugs intended for central nervous system (CNS) disorders, including autism spectrum disorders, bipolar disorder, amyotrophic lateral sclerosis, attention deficit hyperactivity disorder, Duchenne and Becker muscular dystrophy, multiple sclerosis, obsessive–compulsive disorder, migraine, depression, epileptic disorder, Parkinson’s disease, generalized anxiety disorder, social anxiety, panic disorder, schizophrenia, alcohol dependence, post-traumatic stress disorder, smoking, Alzheimer’s disease, insomnia, and premenstrual dysphoric disorder (source: https://www.ema.europa.eu/en/human-regulatory/research-development/scientific-guidelines/clinical-efficacy-safety/clinical-efficacy-safety-nervous-system, accessed on 17 November 2024).

Previously, in 2012, the FDA established the Office of Neuroscience to aid in the development of drugs related to the central nervous system (CNS) (source: https://www.fda.gov/about-fda/center-drug-evaluation-and-research-cder/office-neuroscience, accessed on 17 November 2024). The FDA’s Office of Neuroscience (ON) comprises five distinct review divisions:

- The Division of Neurology oversees the evaluation of new applications for drug and biologic products targeting neurodegenerative disorders, movement disorders, and neuromuscular disorders. This includes conditions such as Alzheimer’s disease, Parkinson’s disease, and Huntington’s disease.
- The Division of Neurology II is responsible for assessing new drug applications concerning seizures, epilepsies, medical countermeasures, migraine, other types of headaches, traumatic brain injuries, inner ear disorders, stroke, and neuroimmunological disorders (e.g., multiple sclerosis).
- The Division of Psychiatry manages applications for biological candidates intended to treat psychiatric diseases and conditions. This encompasses disorders like attention deficit hyperactivity disorder (ADHD), bipolar disorder, schizophrenia, major depressive disorder, obsessive–compulsive disorder (OCD), panic attacks, post-traumatic stress disorder (PTSD), generalized anxiety disorder, autism spectrum disorder, and insomnia.
- The Division of Anesthesia, Addiction Medicine and Pain Medicine (DAAP) oversees and reviews new applications for drug and biological products used in various pain scenarios, including acute and chronic pain. Additionally, it regulates drugs related to addiction (e.g., nicotine, alcohol, stimulants, and opioids) and drug product classes utilized in surgical, procedural, or intensive care unit (ICU) settings to provide patient comfort and ease of treatment. This includes general anesthetics, local anesthetics, dental anesthetics, topical anesthetics, neuromuscular-blocking agents, and agents for reversing neuromuscular blockade.
- The Division of Drug/Toxicology for Neuroscience (DPT-N) also plays a crucial role in this landscape.

## 2. Materials and Methods

To develop our paper, we first referred to the EQUATOR NETWORK, which, while comprehensive, is not very user-friendly for beginners. Next, we examined the websites of key regulatory authorities (Table 1) and supplemented our research with the latest academic papers. For information on pharmaceutical packaging, we utilized platforms that help locate companies in the pharmaceutical packaging sector, especially those involved in clinical trials. For instance, ClinicalTrials.gov offers insights into clinical trials and the associated companies. Additionally, platforms like Pharmaceutical Technology, Packaging Europe, and CPhI Worldwide provide directories and information on suppliers of pharmaceutical packaging solutions for clinical trials. Europages and Kompass are global B2B directories that also assist in finding these specialized companies, and PharmOut offers consultancy services related to pharmaceutical packaging, including support for clinical studies.

## 3. Results

The aim of this brief manuscript is to describe a CNS clinical trial and delineate the procedures required to obtain authorization.

Clinical studies are typically conducted in three phases before gaining marketing approval: Phase I, aimed at assessing safety, determining a safe dosage range, and identifying potential side effects; Phase II, for further safety evaluation and assessing effectiveness; and Phase III, to confirm safety and effectiveness, but also to monitor side effects [9]. The initial step in a clinical study involves clearly defining all research phases and drafting a document elucidating the objectives, duration, and number of patients involved. Subsequently, it is imperative to develop an experimental protocol encompassing several critical elements, including the study’s rationale, primary objectives, specific methodologies employed, data management and analysis strategies, ethical considerations, and approaches to addressing gender-related issues. CNS protocols commence by outlining the fundamental reasons for conducting a particular clinical study within the realm of the central nervous system, elucidating the research’s significance and its potential impact on understanding neurological disorders or therapies. Precisely defined objectives are pivotal in CNS protocols. They provide a precise roadmap of what the research aims to achieve, whether exploring a new treatment’s efficacy, comprehending a neurological disease’s progression, or evaluating intervention impacts. The methodology section illustrates the research’s conduct, encompassing details on data collection methods, patient recruitment, experimental procedures, and any specialized tools or assessments utilized. Effective data management and analysis are foundational in CNS research. This protocol segment delineates data collection, storage, and analysis methods, ensuring the accuracy and reliability of study outcomes. CNS experimental protocols must meet good clinical practice standards and are essential for approval by local ethical committees [10]. A key role in promoting rigorous and transparent reporting practices, thereby elevating the overall quality of health research, can be played by the Equator Network, a global initiative committed to promoting quality and transparency in health research through its meticulously developed guidelines. Its most important contributions are the CONSORT (Consolidated Standards of Reporting Trials) guidelines, which provide a set of guidelines for reporting clinical trial findings transparently and comprehensively, and the STROBE (STrengthening the Reporting of OBservational studies in Epidemiology) guidelines, which play a role in improving reporting for observational study guidelines. These guidelines provide a comprehensive framework designed to advance the reporting standards of clinical trials and observational studies, respectively. Equator’s guidelines contribute significantly to the credibility and reliability of scientific evidence in health research by providing researchers with structured protocols for transparently presenting methodologies, results, and conclusions.

In the design of a CNS clinical study, several rules need to be considered, particularly pertaining to (i) subject selection, (ii) subject evaluation, (iii) variables under study, and (iv) the statistical methodologies used for their analysis [11,12]. Firstly, (i) it is crucial to establish clarity concerning the subjects to be included in the study, selecting patients with similar characteristics while excluding those displaying clinical symptoms that might distort the results [11,12]. Subsequently, (ii) subject evaluation is carried out through a methodology that permits researchers to test the hypothesis. This can involve an objective method employing tools or drugs [12,13]. (iii) The measures considered during the study can either be objective, such as gait speed or response to an electrophysiological stimulus, or subjective, including ratings on evaluation scales or responses to subjective questionnaires [12]. Finally, (iv) the choice of statistical analyses must be carefully made to suit the characteristics of the variables requiring characterization, taking into account the role of researchers in the study (i.e., whether the study is open, blind, or double-blind). Therefore, the actual statistical analysis draws upon personal knowledge enriched by specialists who evaluate clinical variables [12,13] (source: https://www.ema.europa.eu/en/documents/scientific-guideline/ich-e-8-general-considerations-clinical-trials-step-5_en.pdf, accessed on 17 November 2024). The estimation of the number of patients should align with the restrictive parameters set by power analysis (a statistical method to determine the sample size needed to detect a meaningful effect). Inclusion and exclusion criteria must be considered for enlistment, as well as any criteria for the removal or replacement of subjects. Furthermore, an additional 5–10% of participants should be estimated to account for potential adverse effects during the procedure. Notably, software tools, such as https://clincalc.com/stats/samplesize.aspx (accessed on 17 November 2024), can assist in obtaining the specific parameters for the actual sample size. Details regarding the estimated number of recruited subjects, recruitment criteria, recruitment methods, and any group divisions along with drug administration procedures should be explicitly outlined [11,14]. 

Subsequently, it is crucial to specify the procedure for overseeing the entire process, delineate the available support for subjects, establish the means of contact, and define the expected response time. In addition, researchers must outline protocols for managing emergencies and addressing deferred difficulties, with a focus on prioritizing the well-being of the study participants [15]. An observation period subsequent to the trial should be projected for each participant. Comprehensive health insurance coverage is imperative for the entire duration of the study and subsequent follow-up period (Registries for Evaluating Patient Outcomes: A User’s Guide [Internet]. 3rd edition. https://effectivehealthcare.ahrq.gov/sites/default/files/pdf/registries-guide-3rd-edition_research.pdf, accessed on 17 November 2024).

The document should encompass details regarding the frequency of scheduled visits and specify the nature of evaluations, both instrumental and non-instrumental, to be conducted on the patient.

Ultimately, it is the duty of the researcher to enroll the approved clinical trial in the clinical trial registration system managed by the government or an accredited institution. For instance, ‘ClinicalTrials.gov’ serves as a comprehensive repository containing records and outcomes from both privately and publicly funded clinical trials conducted globally, overseen by the US National Library of Medicine. Each clinical trial registration system offers comprehensive guidance and instructions crucial for submitting the necessary documents to the ethics committee (https://classic.clinicaltrials.gov/ct2/manage-recs/present, accessed on 17 November 2024).

The final stage in composing the document involves detailing the following: (i) the maximum anticipated benefits derived from the study, (ii) the procedures intended to mitigate potential harm to the involved parties, (iii) the anticipated adverse effects and corresponding rescue measures, (iv) potential future resources in the event of study success, and (v) the minimum resources permissible in case of study failure.

The study culminates with the comprehensive refinement of all design components encompassing subjects, methodologies, and statistical procedures. Subsequently, this document necessitates submission for approval to the ethical committee (source: https://health.ec.europa.eu/system/files/2020-02/12_ec_guideline_20060216_en_0.pdf, accessed on 17 November 2024). 

The selection of the ethical committee for trial presentation involves a specific procedure. Ethics Committees or Institutional Review Boards (IRBs) are typically selected based on the trial’s geographical location. If the hospital or clinical site designated for the trial and subject recruitment lacks an internal ethics committee, reference will be made to an external reference committee or the one designated for the local headquarters or administrative health area with jurisdiction. The initial step involves preparing documentation and submitting a written request for its approval. Typically, the presentation to the ethical committee encompasses health insurance coverage for the study. Should this coverage not be included, researchers would need to seek an insurance company specializing in clinical trials. This insurance should offer compensation in cases of physical harm incurred during the trial. Contract Research Organizations (CROs) commonly provide this service. However, should researchers prefer an independent approach, contacting a reputable insurance company with a local entity specializing in this process is adequate.

Furthermore, it is imperative to establish contact with a pharmaceutical company, a licensed pharmacy, or an authorized entity for drug packaging. Researchers must ensure that the drugs are accompanied by the package leaflet of the medical product. Numerous multinational corporations and non-CROs offer services for drug packaging and the associated documentation process (source: https://www.catalent.com/; https://stmpharmapro.it/it/; https://stmpharmapro.it/it, accessed on 17 November 2024). 

The documentation ought to encompass the following: (i) details regarding the manufacturing site, including bulk production, primary packaging, secondary packaging, quality control, and batch certification; (ii) manufacturing authorization (MIA); and other (iii) specifications pertaining to control processes involved in drug procedures.

It is also feasible to enlist a sponsor for the clinical trial, as recommended by the FDA guideline (https://www.fda.gov/media/75398/download, accessed on 17 November 2024). 

Upon competition of all necessary authorizations and documents, in cases where a sponsor is absent and there is an intent to repurpose a substance that has already undergone the four experimental stages, researchers need only focus on determining the method of drug administration. This stage requires a meticulous assessment of potential statistical permutations occurring between the drug and placebo, considering diverse patient cohorts stratified by variables such as age, sex, socioeconomic status, and underlying pathological conditions. To ensure the avoidance of potential errors and establish an effective strategy for substance administration, it is essential to devise a straightforward compound that is easily recognizable and administrable. To achieve this objective, researchers must implement a system for labeling and randomizing the drug versus placebo among the various subgroups of enrolled subjects. The labels, progressively numbered barcodes, should contain information identifying the patient, the specific year and month of substance administration, and the type of substance administered. This process should be conducted without the researcher having access to detailed information but under their supervision to indicate potential permutations.

## 4. Discussion

This paper outlines the critical steps and considerations regarding clinical trials’ design and executive steps, specifically in the context of central nervous system (CNS) studies. The process for the clinical trial is structured into four phases (P): P-I is fixed on safety and dosage assessment, P-II requires additional safety evaluations and effectiveness testing, and P-III is conducted to confirm the safety and efficacy of the used treatment while monitoring side effects. P-IV is the post-marketing surveillance after treatment has received regulatory approval and is available for public use. Each phase charges a clear and well-defined research protocol, expressing objectives, duration, participant selection criteria, and methodologies.

An essential aspect of designing clinical studies, particularly CNS trials, is the careful development of a protocol. The protocol should describe the study’s rationale and objectives, including data collection methods and ethical and gender-related issues.

It is also necessary to ensure the operation of data management strategies and experimental procedures, which are crucial for obtaining accurate and reliable results. Additionally, adhering to ethical standards and regulatory requirements, such as good clinical practice (GCP) guidelines and obtaining ethical committee approval, is fundamental for safeguarding the enrolled subject and ensuring the success of the clinical study.

We want to emphasize the importance of clear subject selection criteria. Evaluating participants is an essential step in testing a study’s hypotheses. Appropriate statistical tests and methods must also be used to analyze the data about the study variables and stratify the data using basal variables. In this context, it is crucial that the role of power analysis before starting the study is to determine the correct sample size. The right sample size can contribute to reducing errors and providing excellent reliability for the study.

In addition to these methodological aspects, researchers are also responsible for ensuring patient safety, including addressing potential adverse effects and offering health insurance coverage for participants during the study and follow-up period. Ethical committees’ approval and oversight of the study are essential steps in ensuring the trial’s adherence to ethical and regulatory standards.

The final stages of the trial concern the preparation for drug packaging, labeling, and randomization. Usually, this step is realized by the pharmaceutical company if the trial is sponsored. However, when the study is realized only on scientific intuition without a sponsor, it is necessary to involve a service company. If it is impossible to dedicate funding for this process, realize the procedure steps, following the law disposition of regulatory authorities. This step is essential for maintaining the integrity of the study and minimizing bias.

During the trials, researchers are responsible for correct drug administration, whether a new or a repurposed substance is correctly administered, and all trial steps are conducted transparently and according to regulatory guidelines.

In summary, successful clinical trials require meticulous planning, clear protocols, ethical considerations, and rigorous statistical analysis. In this contest, we have created this rapid checklist for beginners that can help in the design of trials during the first project steps.

Researchers must navigate multiple regulatory and logistical challenges to ensure the study’s success and produce reliable, valid results that can advance medical knowledge and treatment options

## 5. Conclusions

This manuscript aims to serve as a foundational resource for recently graduated students or novice researchers seeking to understand the essentials of conducting a CNS clinical study, accounting for the intended type of study and prevailing regulatory frameworks. The abundance of information available on the internet can overwhelm readers, particularly those in the early stages of their research careers who require a comprehensive guide for their experimentation. To address this need, we have curated a concise list to provide clear direction in structuring a clinical study, aiding in a better understanding of its fundamental aspects. It is important to note that this manuscript does not aspire to be exhaustive; it serves as a repository of valuable insights for guidance and reference purposes.

## Figures and Tables

**Table 1:** Major authorities for clinical trials. Authorities for laws and protocols in clinical trials include global and national organizations that regulate clinical studies’ safety, efficacy, and ethics. The table includes also web referments. Light gray brings together the entire agency under the control of EMA; gray indicates the whole agency in Asian territories, while little light gray indicates the agencies of south America, and in the last color are indicate all the remaining countries.

Country	Regulatory Body	Actually Under Control of
Austria	Austrian Agency for Health and Food Safety	The European Medicines Agency (EMA) 2022 https://www.ema.europa.eu/en (accessed on 17 November 2024)
Belgium	Federal Agency for Medicines and Health Products (FAMHP)	
Bulgaria	Bulgarian Drug Agency	
Croatia	Agency for medicinal products and medical devices of Croatia	
Cyprus	Ministry of Health—Pharmaceutical Services	
Czechia	State Institute for Drug Control	
Denmark	Danish Medicines Agency	
Estonia	State Agency of Medicines	
Finland	Finnish Medicines Agency	
France	National Agency for the Safety of Medicine and Health Products	
Germany	Federal Institute for Drugs and Medical Devices	
Greece	National Organization for Medicines	
Hungary	National Institute of Pharmacy and Nutrition	
Iceland	Icelandic Medicines Agency	
Ireland	Health Products Regulatory Authority (HPRA)	
Italy	Italian Medicines Agency	
Latvia	State Agency of Medicines	
Liechtenstein	Office of Health / Department of Pharmaceuticals	
Lithuania	State Medicines Control Agency	
Luxembourg	Ministry of Health	
Malta	Malta Medicines Authority (MMA)	
Netherlands	Medicines Evaluation Board Healthcare and Youth Care Inspectorate, Ministry of Health, Welfare and Sport	
Norway	Norwegian Medicines Agency	
Poland	Office for Registration of Medicinal Products, Medical Devices and Biocidal Products	
Portugal	National Authority of Medicines and Health Products	
Romania	National Authority of Medicines and Medical Devices of Romania	
Slovakia	State Institute for Drug Control	
Slovenia	Agency for Medicinal Products and Medical Devices of the Republic of Slovenia	
Spain	Spanish Agency for Medicine and Medical Devices	
Sweden	Swedish Medical Products Agency	
Bangladesh	Directorate General of Drug Administration (DGDA)	http://dgdagov.info/ (accessed on 17 November 2024)
China	State Food and Drug Administration (SFDA)	http://www.sfda.com/ (accessed on 17 November 2024)
	National Medical Products Administration (NMPA)	http://english.nmpa.gov.cn (accessed on 17 November 2024)
Hong Kong	Drug Office—Department of Health	https://www.dh.gov.hk/english/main/main_ps/main_ps.html (accessed on 17 November 2024)
India	Central Drug Standards Control Organization (CDSCO)	https://cdsco.gov.in/opencms/opencms/en/Home/ (accessed on 17 November 2024)
Japan	Ministry of Health, Labor and Welfare (MHLW)	https://www.mhlw.go.jp/english/ (accessed on 17 November 2024)
	Japanese Pharmaceuticals and Medical Devices Agency (PMDA)	https://www.pmda.go.jp/english/ (accessed on 17 November 2024)
Malaysia	Ministry of Health (MOH)	https://www.moh.gov.my/ (accessed on 17 November 2024)
Philippines	Department of Health (DOH)	https://lawphil.net/administ/doh/doh.html (accessed on 17 November 2024)
Singapore	Health Sciences Authority (HSA)	https://www.hsa.gov.sg/ (accessed on 17 November 2024)
South Korea	Ministry of Food and Drug Safety (MFDS)	https://www.mfds.go.kr/eng/index.do (accessed on 17 November 2024)
Thailand	Food and Drug Administration of Thailand (FDATA)	https://en.fda.moph.go.th/ (accessed on 17 November 2024)
Taiwan	Taiwan Food and Drug Administration (TFDA)	https://www.fda.gov.tw/eng/ (accessed on 17 November 2024)
Vietnam	Drug Administration of Vietnam	https://dav.gov.vn/dich-vu-cong-ce5.html (accessed on 17 November 2024)
Brazil	National Health Surveillance Agency (ANVISA)	The HHS Office for Human Research Protections (2021) https://www.hhs.gov/ohrp/index.html (accessed on 17 November 2024)
Colombia	Ministry of Health	
Perú	Ministry of Health	
Chile	Ministry of Health	
Canada	CanadaFDA, Health Canada (HC) reviews	https://www.canada.ca/en/health-canada/services/drugs-health-products.html (accessed on 17 November 2024)
USA	The United States Food and Drug Administration (FDA or US FDA)	https://www.fda.gov/ (accessed on 17 November 2024)
New Zealand	Medsafe is the Medicines and Medical Devices Regulatory Authority	https://www.medsafe.govt.nz/ (accessed on 17 November 2024)
Australia	The Therapeutic Goods Administration (TGA)	https://www.tga.gov.au/ (accessed on 17 November 2024)

## Data Availability

All the data shown in this paper are available in the PubMed Library. The authors created all representative illustrations where appropriate, which are available on request.

## Supplementary Materials

The following supporting information can be downloaded at https://www.mdpi.com/article/10.3390/neurolint17010001/s1. Figure S1: Types of Clinical studies. References [16,17,18,19] are cited in the supplementary materials.
