# Supplementary material for: Leaflet: Operative Steps for Interventional Studies in Neuroscience
Source: Neurol Int. 2024 Dec 24;17(1):1. doi: 10.3390/neurolint17010001 (PMC11767703; doi:10.3390/neurolint17010001)
Supplement: Supplementary file 1 [file neurolint-17-00001-s001.zip › neurolint-3350608-supplementary.pdf]

Preclinical development

Preclinical development, also referred to as preclinical studies or non-clinical studies, constitutes a pivotal research phase in drug development that precedes human clinical trials. Throughout this stage, researchers collect essential data to assess the viability of the drug and conduct numerous tests to ensure its safety [1].

Initially, this phase is carried out in vitro, particularly on cell cultures or in silico organs, and subsequently on laboratory animals, starting with simpler species and progressively advancing to small mammals within the rodent family [1]. Preclinical studies involving animals play a crucial role in evaluating the effectiveness of therapeutic drugs and strategies before advancing to clinical trials. However, it's important to note that discussing these studies in detail is beyond the scope of this work.

While in vitro preclinical studies are not subject to specific legislation, studies involving animals in vivo demand careful consideration of the animals as sentient beings. They are subject to various laws and regulations designed to safeguard the well-being of the animal species (Figure 1).

Overall, each clinical study must be planned carefully since every stage plays a vital role in the quality, execution, and interpretation of biomedical research [8]. The choice of the study design is a crucial part of the study goal,relying on many variables, including previous research, availability of study enrollees, funding, and time limitations. In general, two main types of clinical research can be identified: i) observational studies and ii) interventional studies. Other types of clinical studies are summarized in Figure S1.

Discovery phase to preclinical development

The transition from discovery to preclinical development in neuroscience drug development includes key stages for identifying and optimizing promising therapeutic candidates. Early results are essential for selecting and refining compounds for clinical trials.

Discovery Phase

The discovery phase identifies neurological targets, such as receptors, enzymes, or signalling pathways involved in diseases like Alzheimer's, Parkinson's, or depression. High-throughput screening (HTS) is utilized to identify potential compounds that interact with these targets. It's important to note that the blood-brain barrier, a highly selective semipermeable membrane that separates the circulating blood from the brain and extracellular fluid in the central nervous system, presents a significant challenge in drug development for neurological diseases. This is followed by optimization of these candidates for potency, selectivity, and pharmacokinetic properties [16].

Preliminary results from in vitro assays are vital. Initial screenings identify active compounds; selectivity assays help eliminate compounds with off-target effects, and toxicity testing at early stages flags potentially harmful candidates [17]. For instance, a small molecule that targets a neuroinflammatory pathway could be discarded if early assays reveal it has non-selective activity or toxicity to neuronal cells [18].

Example: A compound designed to inhibit a specific kinase involved in neurodegeneration may show activity in cell-based assays, but if initial data reveal significant neurotoxicity at therapeutic concentrations, it may be abandoned or re-engineered to reduce such effects [18].

Lead Optimization

Once promising hits are identified, the lead optimization phase, a crucial stage in drug development, refines these molecules to improve their pharmacokinetics, bioavailability, and stability, ensuring they are suitable for in vivo testing. This phase involves a series of iterative steps, including structural modifications, to enhance the drug's properties. Pharmacodynamics (PD) and pharmacokinetics (PK) studies are then con-

ducted to determine how the compound interacts with its target and behaves within the body [19].

Preliminary in vivo data provide essential information regarding the compound's safety and efficacy. These results are crucial in determining the future of the compound. If a compound shows promise but induces liver toxicity or crosses the blood-brain barrier too slowly, structural modifications are made to improve its metabolic stability or CNS penetration [18].

Example: A candidate compound that effectively inhibits a receptor implicated in Parkinson's disease may be cleared too quickly from the body, thus limiting its efficacy. In this case, the compound's chemical structure may be modified to improve its pharmacokinetics and extend its half-life, enhancing its therapeutic potential [19].

Preclinical Development

In the preclinical development phase, candidates undergo more rigorous testing in reliable animal models to evaluate their safety, efficacy, and pharmacokinetic profiles. This phase includes repeated-dose toxicology studies and efficacy evaluations in models of neurodegenerative diseases, such as rodent models of Alzheimer's or Parkinson's disease, to confirm the compound's therapeutic potential [16].

Preliminary results from toxicology studies provide essential data on dose-dependent toxicity and organ-specific effects, which help establish an appropriate dosing regimen for clinical trials. Additionally, efficacy data from animal models confirm whether the compound can elicit the desired therapeutic effects in vivo [17].

Example: A compound showing promise in reducing neuroinflammation in a mouse model of Alzheimer's may be advanced to preclinical testing. However, if high doses cause toxicity to the liver or other organs, further dose optimization and safety studies would be necessary before progressing to human clinical trials [18].

Throughout the drug development process in neuroscience, preliminary results from in vitro and in vivo studies serve as essential guides for selecting and optimizing promising candidates.

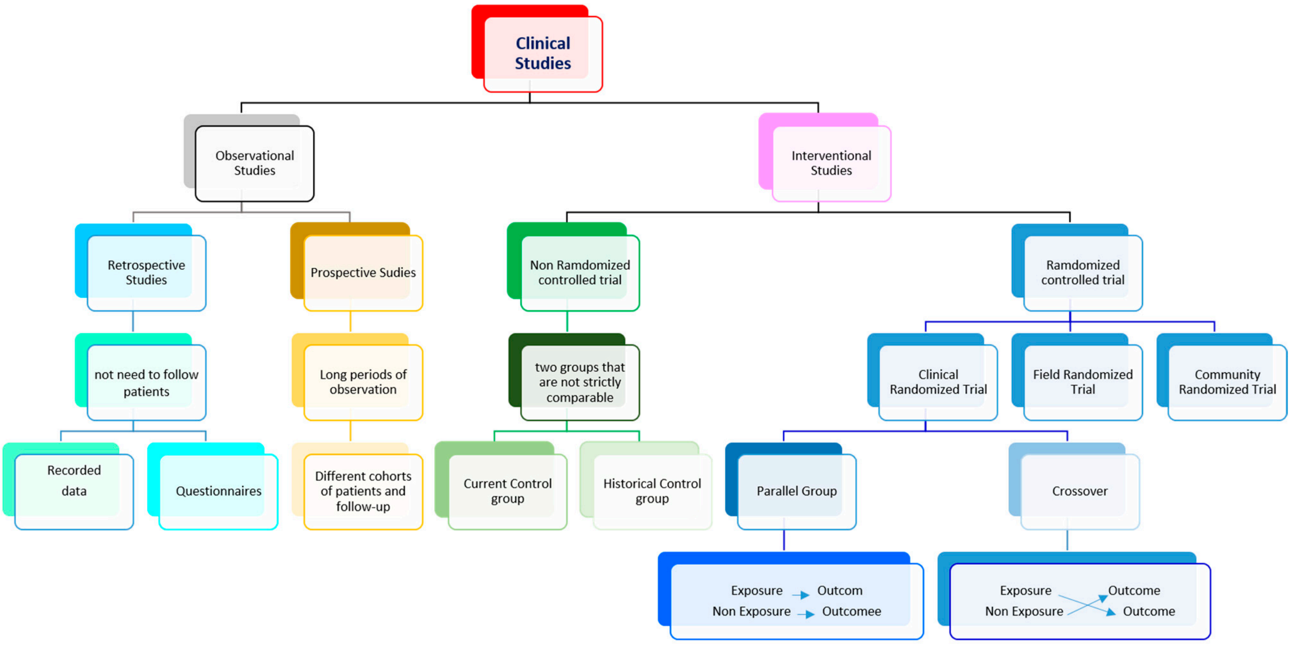

Figure S1. Types of Clinical studies. The flowchart outlines the main categories of clinical trials, which include interventional studies, observational studies, and expanded access studies. It is essential to choose the appropriate type of study to ensure both scientific validity and clinical relevance. The study design should align with the research question, the target population, and the outcome measures. Regulatory authorities such as the FDA, EMA, and WHO have different protocols for each type of trial to monitor progress and ensure compliance with ethical, safety, and efficacy standards. Selecting the right protocol is important for accurate data collection and meaningful results, which in turn support thorough regulatory review and approval processes. support thorough regulatory review and approval processes.
